# Functional Insights into the Sphingolipids C1P, S1P, and SPC in Human Fibroblast-like Synoviocytes by Proteomic Analysis

**DOI:** 10.3390/ijms25158363

**Published:** 2024-07-31

**Authors:** Thomas Timm, Christiane Hild, Gerhard Liebisch, Markus Rickert, Guenter Lochnit, Juergen Steinmeyer

**Affiliations:** 1 Protein Analytics Group, Institute of Biochemistry, Justus Liebig University Giessen, 35392 Giessen, Germany; 2 Laboratory for Experimental Orthopaedics, Department of Orthopaedics, Justus Liebig University Giessen, 35392 Giessen, Germany; 3 Institute of Clinical Chemistry and Laboratory Medicine, University Hospital Regensburg, 93053 Regensburg, Germany

**Keywords:** ceramide-1-phosphate, sphingosine-1-phosphate, sphingosylphosphorylcholine, lysosphingomyelin, sphingolipid, proteomics, inflammation, mass spectrometry, functions, osteoarthritis

## Abstract

The (patho)physiological function of the sphingolipids ceramide-1-phosphate (C1P), sphingosine-1-phosphate (S1P), and sphingosylphosphorylcholine (SPC) in articular joints during osteoarthritis (OA) is largely unknown. Therefore, we investigated the influence of these lipids on protein expression by fibroblast-like synoviocytes (FLSs) from OA knees. Cultured human FLSs (*n* = 7) were treated with 1 of 3 lipid species—C1P, S1P, or SPC—IL-1β, or with vehicle. The expression of individual proteins was determined by tandem mass tag peptide labeling followed by high-resolution electrospray ionization (ESI) mass spectrometry after liquid chromatographic separation (LC-MS/MS/MS). The mRNA levels of selected proteins were analyzed using RT-PCR. The 3sphingolipids were quantified in the SF of 18 OA patients using LC-MS/MS. A total of 4930 proteins were determined using multiplex MS, of which 136, 9, 1, and 0 were regulated both reproducibly and significantly by IL-1β, C1P, S1P, and SPC, respectively. In the presence of IL-1ß, all 3 sphingolipids exerted ancillary effects. Only low SF levels of C1P and SPC were found. In conclusion, the 3 lipid species regulated proteins that have not been described in OA. Our results indicate that charged multivesicular body protein 1b, metal cation symporter ZIP14, glutamine-fructose-6-P transaminase, metallothionein-1F and -2A, ferritin, and prosaposin are particularly interesting proteins due to their potential to affect inflammatory, anabolic, catabolic, and apoptotic mechanisms.

## 1. Introduction

Osteoarthritis (OA) is a chronic, destructive, and painful joint disease that has been associated with an abnormal metabolism of joint lubricants. The synovial fluid (SF) of OA patients bears altered joint lubricant profiles, containing increased amounts of lubricin, hyaluronan, and phospholipids (PLs) [1,2,3]. PLs in the SF originate from fenestrated synovial blood vessels and type B, or fibroblast-like synoviocytes (FLSs) [4]. In a prior study with cultured human OA FLSs, we demonstrated that transforming growth factor-ß1 (TGF-ß1) and insulin-like growth factor-1 (IGF-1) induce phosphatidylcholine (PC) biosynthesis, whereas interleukin-1ß (IL-1ß) upregulates phosphatidylethanolamine and plasmalogens [5,6]. Further, IL-1ß stimulated the release of PC by increasing the expression of ABCA1 transporters in OA FLSs [4]. Notably, IL-1ß concentrations in the SF peaked in early knee OA (KL score I) and decreased 4 to 5-fold in advanced stages (KL grade II–IV) [7,8].

Despite these insights, the functions of PLs in articular joints remain inadequately examined. Using mass spectrometry (MS)-based proteomics, our recent study revealed distinct protein expression profiles in human FLSs that were induced by three slightly differing LPC species, of which LPC 16:0 was the most active [9]. Prompted by these findings, our focus shifted to ceramide-1-phosphate (C1P), sphingosine-1-phosphate (S1P), and sphingosylphosphorylcholine (SPC) in the current proteomic study.

C1P, a ceramide metabolite, is formed through ceramide phosphorylation by ceramide kinase (CerK), primarily with substrate specificity for C16 ceramides. CerK can be stimulated by IL-1ß and is associated with the trans-Golgi network [10]. Unlike S1P, C1P is not secreted but is released by leaky or damaged cells and exported to the plasma in exosomes to interact with other cell membranes [10]. In mice, C1P concentrations of 3.6, 4.9, and 45 pmol/mg protein were determined in neutrophils, mast cells, and macrophages, respectively [11]. Plasma from healthy normo-lipidemic volunteers contains 62 + 20 nM of C1P 18:1;O2/16:0 [12]. As a low-affinity agonist, C1P has been reported to bind to a possible Gi protein-coupled receptor in macrophages [13]. Whether cells in the articular joint harbor such a receptor is unknown.

C1P is a multifaceted molecule with diverse functions, some of which are cell-specific and distinct from those of other sphingolipid metabolites [10,14,15,16,17]. Balancing the concentrations of C1P and ceramide is crucial for cell and tissue homeostasis and the “sphingolipid rheostat” governs this equilibrium with S1P. C1P regulates cell proliferation and survival [16] and, like S1P, is a potent inhibitor of apoptosis, whereas ceramides are proapoptotic [15]. In plasma, C1P is believed to participate in the recruitment of stem/progenitor cells to damaged organs and to stimulate vascularization. Moreover, C1P mediates inflammation by promoting the release of arachidonic acid through the activation of cytosolic phospholipase A2α (cPLA2α), a critical enzyme in inflammatory prostaglandin and leukotriene production [18,19,20]. C1P can synergize with S1P, based on the ability of S1P to upregulate cyclooxygenase-2 (COX-2) [15]. C1P also has anti-inflammatory effects in certain cells and tissues, such as macrophages and lung tissue. For instance, it inhibits the lipopolysaccharide (LPS)-induced production of IL-6, IL-8, and IL-1ß in human peripheral blood mononuclear cells [21]. The potential impact of these effects in articular joints merits further examination.

S1P, a crucial element in intracellular and intercellular signaling, arises from newly synthesized ceramide or as part of the sphingomyelin cycle or sphingolipid rheostat [22]. S1P binds avidly to G-protein-coupled S1P receptors (S1PR1 to S1PR5), with downstream targets varying between tissues [22]. S1PR1 has been observed during RA and OA in synovial lining cells, vascular endothelial cells, and inflammatory mononuclear cells of the synovium [23]. Notably, fingolimod, a modulator of the S1P receptor and an established therapeutic agent in multiple sclerosis [24], has therapeutic potential for bone-related diseases [25].

Most S1P in blood is produced by erythrocytes, platelets, mast cells, and monocytes. An MS-based analysis of human plasma from 25 healthy normo-lipidemic subjects revealed a concentration of 530 ± 90 nM for S1P 18:1;O2 and 290 ± 90 nM for S1P 18:0;O2 [12]. Serum reference values for S1P were determined in a cohort of 1339 healthy volunteers, yielding a median of 804 nM and a reference interval of 534–1242 nM (2.5th; 97.5th percentile) [26]. Most plasma S1P is bound to apolipoprotein M, in association with high-density lipoproteins (HDLs) and albumin [27]. 

S1P is a pleiotropic PL that influences cardiac function, vascular development, vascular permeability, immune cell function, cancer, and inflammation [22]. In OA, S1P has been implicated in various processes, including the regulation of apoptosis, PGE2 secretion via COX-2, and VEGF expression [23,28,29,30]. S1PR2 is the predominant S1P receptor subtype in human OA chondrocytes, downregulating aggrecan expression and inhibiting the IL-1ß-induced regulation of ADAMTS-4, MMP-13, and iNOS through the p38 MAPK pathway [31,32,33]. S1P also stimulates chondrocyte proliferation by activating extracellular signal-regulated kinase (ERK) [34]. 

Sphingosylphosphorylcholine (SPC), a multifunctional lysosphingolipid that originates from sphingomyelin, engages in various signal transduction pathways, influencing cellular processes. Bearing structural homology to S1P, SPC can bind as a low-affinity agonist to G-protein-coupled S1P receptors [35] or serve as a second messenger to regulate intracellular calcium release [36]. The impact of SPC on cellular proliferation and differentiation and its proinflammatory and anti-inflammatory properties are challenging to distinguish from those of S1P due to enzymatic conversion by autotaxin [36,37,38]. Released from activated platelets, SPC associates with HDL, with plasma levels of 53 ± 30 nM SPC 18:1;O2 and 9 ± 6 nM SPC 16:1;O2, in healthy normo-lipidemic subjects [12,39]. Elevated SPC levels are observed in such conditions as Niemann–Pick disease type C, metabolic syndrome, and atopic dermatitis [37,38].

The collective data on C1P, S1P, and SPC underscore their diverse and, at times, opposing effects on a cellular level. OA is a disease that affects the entire joint, and ample evidence shows that FLSs undergo significant metabolic changes [40,41]. Our study aims to determine the pathophysiological contribution of important lipids, such as C1P, S1P, and SPC, to these metabolic alterations. Modern proteomic analyses have been instrumental in identifying the intricate functions and interactions of these lipids. Using a recently introduced multiplex proteomic method, several samples were determined by a simultaneous and semi-quantitative mass spectrometry analysis. Specifically, we set out to discern the varying effects of C1P, S1P, and SPC and differentiate them from those that are induced by the proinflammatory cytokine IL-1ß. In addition, the concentrations of these lipids in the SF were quantified by MS, which, other than for S1P, have not been reported. This research constitutes a significant advance in understanding the complex lipid interactions within articular joints, providing valuable insights into the pathophysiology of OA. 

## 2. Results

### 2.1. Regulated Proteins Identified by MS

A total of 4930 master proteins were determined in the extracted FLS using MS. A master protein is a protein with the longest sequence, if more than one protein in a group has the same peptide score, an equal number of peptide spectrum matches (PSMs), and an equal number of peptides. In addition, these key proteins were identified using an FDR of below 0.01, an HT-Sequest score exceeding 30, and the detection of at least one unique peptide for each protein. Furthermore, we excluded contaminants, per the database of Thermo Fisher Scientific. To be included in our analyses as a reproducibly regulated master protein, at least 11 of the 14 replicates in each treatment group had to be stimulated by more than 1.2-fold or downregulated by less than 0.8-fold.

**Figure 1:**
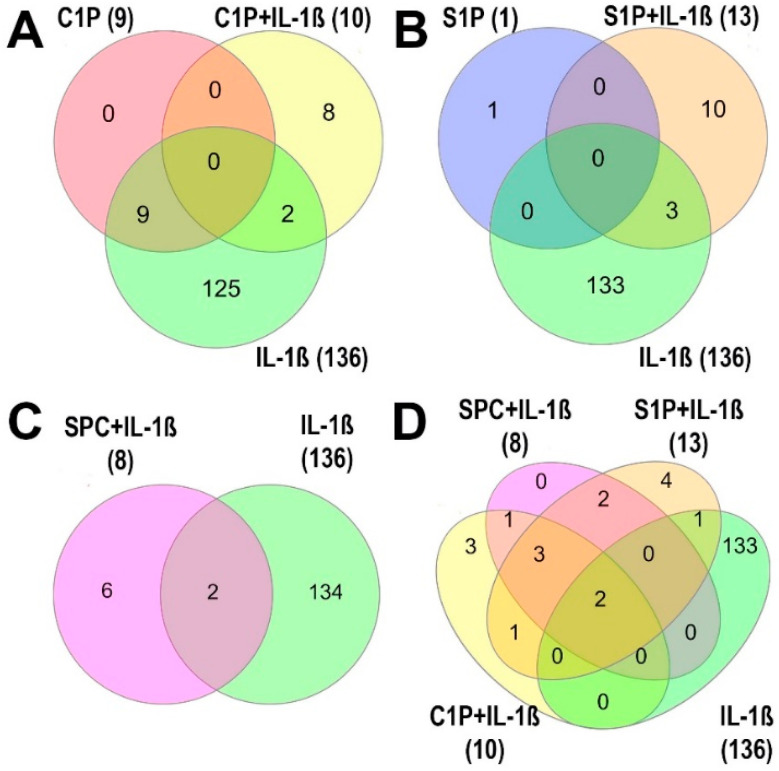
Venn diagram of proteins that are reproducibly regulated by (**A**) C1P (red), C1P in the presence of IL-1ß (yellow), and IL-1ß alone (green); (**B**) S1P (blue), S1P in the presence of IL-1ß (orange), and IL-1ß alone (green); (**C**) SPC in the presence of IL-1ß (violet) and IL-1ß alone (green); and (**D**) IL-1ß in the presence of C1P (yellow), S1P (orange), or SPC (violet). The number of proteins that can be reproducibly regulated is shown. In Tables S1 and S2, the AR of each protein is listed together with the results of the statistical analysis.

**Figure 2:**
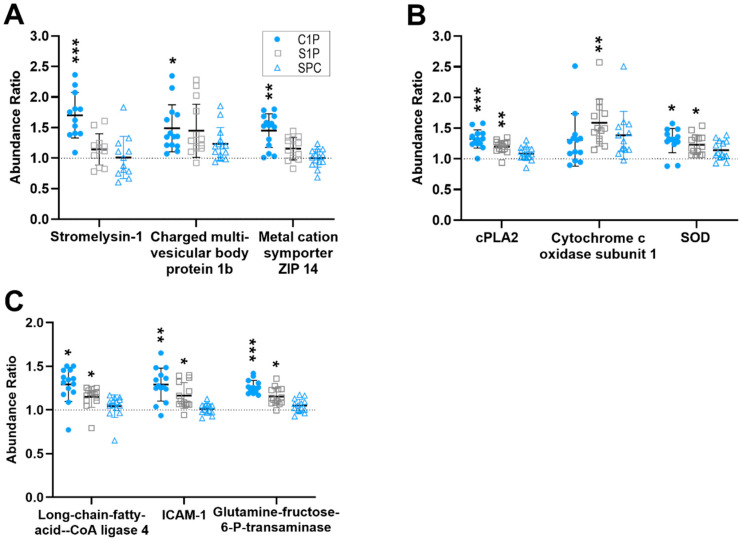
C1P and S1P reproducibly and significantly regulate the level of proteins in FLSs. Protein levels of (**A**) stromelysin, charged multivesicular body protein 1b, metal cation symporter ZIP14; (**B**) cPLA2, cytochrome c oxidase subunit 1, SOD; and (**C**) long-chain-fatty-acid–CoA ligase 4, ICAM-1, and glutamine fructose-6-Ptransaminase were quantified by MS in duplicate in the 7 biological replicates. The dot plots represent the data obtained from the resulting 14 replicates and illustrate the x-fold abundance of the proteins in the treated FLS cells in comparison to that of only vehicle-treated controls (which are normalized to 1 and shown as a dotted line). The mean value ± SD is represented by lines within each figure (*n* = 7). * 0.05 ≥ *p* > 0.01; ** 0.01 ≥ *p* > 0.001; *** *p* ≥ 0.001.

C1P was the most active lipid species that was tested—followed by S1P and SPC—given that it reproducibly and significantly regulated 9 proteins (1 and 0 proteins for S1P and SPC, respectively) (Figure 1). The 9 proteins (P09341, P08254, Q7LBR1, Q15043, P47712, Q7Z7M4, O60488, Q5NKV8, A0A0S2Z4X9) were only upregulated reproducibly by C1P, as was 1 protein (Q4EZA9) by S1P (Figure 2, Table S1). The concentrations of 5 proteins, although unreproducible, were significantly increased (P47712, Q7Z7M4, O60488, Q5NKV8, A0A0S2Z4X9) by S1P, whereas 1 protein (Q4EZA9) was unreproducibly but significantly stimulated by SPC (Figure 2, Table S1). Compared with IL-1ß, the levels of the 9 reproducibly regulated proteins were significantly lower in C1P-treated FLSs, although C1P and IL-1ß reproducibly and significantly upregulated them versus untreated controls (Figure 1, Table S1).

In the presence of IL-1ß, which plays an important role in the pathophysiology of OA, all three lipid species had an additional effect (Figure 1, Table S2). Under these conditions, C1P reproducibly and significantly regulated 10 proteins (P02795, P04733, A0A6I8PLD9, P42677, Q71UM5, A0A024QYX3, P62841, B4DLF7, A0A384MDR3, Q6NS36) compared with IL-1ß-treated controls (Figure 1, Table S2). Moreover, S1P and SPC reproducibly and significantly regulated 13 (P02795, P04733, P09038-1, D6W5K2, Q59EN5, A0A6I8PLD9, O60476, P30048, Q8N5Y3, B4DLF7, Q96FJ2, A0A384MDR3, Q6NS36) and 8 proteins (P02795, P04733, A0A6I8PLD9, A0A024QYX3, P30048, B4DLF7, Q96FJ2, Q6NS36), respectively, with IL-1ß versus IL-1ß alone (Table S2). Similarly, some proteins were significantly but not reproducibly regulated by S1P and SPC in the presence of IL-1ß compared with FLSs that were treated with IL-1ß alone (Table S2). Conversely, IL-1ß, which was added alone, reproducibly regulated 136 proteins (Figure 1). The levels of 119 proteins were consistently higher on average by more than 1.2-fold, and those of 17 proteins decline on average by less than 0.80-fold.

In comparing the ARs of the three lipids in the presence of IL-1ß, they differed markedly in the number of significantly and reproducibly regulated proteins but less so with regard to their levels (Figure 1, Table S2). Thus, in the presence of IL-1ß, only C1P—not S1P or SPC—significantly and reproducibly regulates 3 proteins (P42677, Q71UM5, P62841), and only S1P does so with 5 proteins (P09038-1, D6W5K2, Q59EN5, O60476, Q8N5Y3) (Figure 1, Table S2). In the presence of IL-1ß, SPC also develops a specific expression profile that differs from those of C1P and S1P in terms of the number of significantly and reproducibly regulated proteins (Figure 1, Table S2). 

### 2.2. Regulated Proteins and Their Potential Biological Functions in FLS

**Figure 3:**
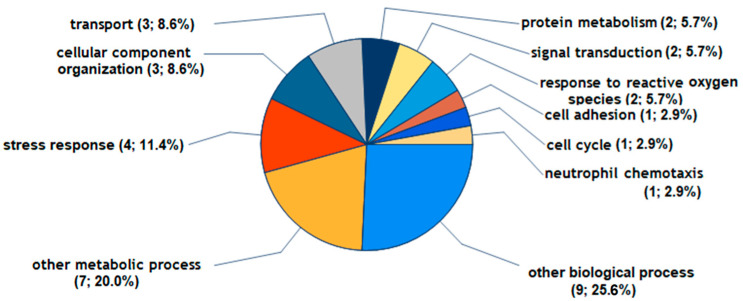
The biological processes of FLS being altered by C1P. The 9 proteins were reproducibly upregulated by more than 1.2-fold by C1P in FLS during 48 h of treatment, and Table S1 provides further data on these proteins. The Go Slim categories for proteins were generated by Proteome Discoverer 2.5 software using the Gene Ontology (GO) database.

According to the GO Slim categories used for annotating biological processes, the majority of the 9 proteins significantly and consistently regulated by C1P are associated with stress response and organization (P09341, P08254, Q7LBR1, Q7Z7M4), cellular component organization (P09341, P08254, Q7LBR1), transport (Q7LBR1, Q15043, P47712), protein metabolism (P08254, Q7LBR1), signal transduction (P09341, Q15043), response to reactive oxygen species (P08254, Q7Z7M4), cell adhesion (Q5NKV8), cell cycle (Q7LBR1), and neutrophil chemotaxis (P09341) (Figure 3). 

**Figure 4:**
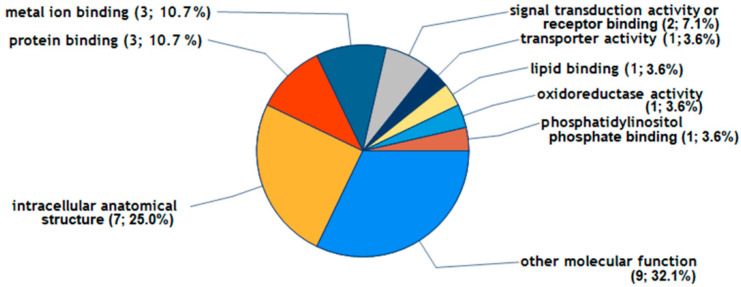
The molecular functions of FLS being altered by C1P. The 9 proteins were reproducibly upregulated by more than 1.2-fold by C1P in FLS during 48 h of treatment, and Table S1 provides further data on these proteins. The Go Slim categories for proteins were generated by Proteome Discoverer 2.5 software using the Gene Ontology (GO) database.

For the annotation of molecular functions, C1P modulated the level of proteins that participate in intracellular anatomic structure (P09341, P08254, Q7LBR1, Q15043, P47712, Q7Z7M4, O60488), protein binding (P09341, Q7LBR1, Q5NKV8), metal ion binding (P08254, P47712, Q7Z7M4), and signal transduction (P09341, Q5NKV8) (Figure 4).

**Figure 5:**
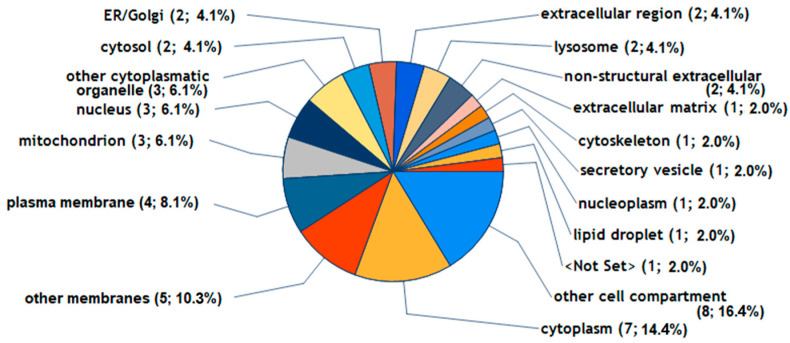
The cellular localization of 9 proteins being regulated by C1P. The 9 proteins were reproducibly upregulated by more than 1.2-fold by C1P in FLS during 48 h of treatment, and Table S1 provides further data on these proteins. The Go Slim categories for proteins were generated by Proteome Discoverer 2.5 software using the Gene Ontology (GO) database.

The differentially expressed proteins that were induced by C1P resided in several cellular structures, including parts of the cytoplasm (P09341, P08254, Q7LBR1, Q15043, P47712, Q7Z7M4, O60488), other membranes (Q7ZLBR1, Q15043, P47712, O60488, Q5NKV8), plasma membrane (Q7LBR1, Q15043, O60488, Q5NKV8), mitochondria (P47712, Q7Z7M4, O60488), nucleus (P08254, Q7LBR1, P47712), other cytoplasmatic organelles (Q7LBR1, Q15043, O60488), cytosol (Q7LBR1, P47712), ER/Golgi (P47712, O60488), extracellular regions (P09341, P08254), lysosomes (Q7LBR1, Q15043), and the nonstructural extracellular (P09341) and extracellular matrix (P08254) (Figure 5).

S1P significantly and reproducibly regulated only 1 protein (Q4EZA9, cytochrome c oxidase subunit 1), which is involved in transport and other metabolic and biological processes, with its molecular functions including transporter activity, intracellular anatomical structure, oxidoreductase activity, and metal ion binding. Q4EZA9 has been described as a cellular component of mitochondria, the cytoplasm, and other cell compartments and membranes. 

IL-1ß reproducibly regulated 136 proteins by more than 1.2-fold or less than 0.8-fold on average. All 9 proteins being significantly and reproducibly modulated by C1P were also consistently regulated by IL-1ß (Figure 1, Table S1). Notably, the three lipid species had additional stimulatory or inhibitory effects on protein expression in FLSs that were treated with IL-1ß. As such, 2 of 10 proteins (P02795, P04743) were consistently and markedly regulated by C1P in the presence of IL-1ß, similar to FLSs treated solely with IL-1ß, albeit lacking reproducibility; however, the expression of the other eight proteins were reproducibly stimulated or inhibited only by C1P in the presence of IL-1ß but not by IL-1ß alone (Table S2). Thus, the affected biological processes, molecular functions, and location within cellular structures of proteins of FLSs that were treated with C1P in the presence of IL-1ß differed markedly from those that were identified by the applied GO Slim categories for C1P that was administered alone (Figures S1–S3). 

Again, only 3 (O60476, P02795, P04733) of 13 proteins that were consistently and significantly regulated were stimulated by S1P in the presence of IL-1ß, which is similar to the FLS treated solely with IL-1ß but not reproducibly (Table S2). However, only 1 protein (Q4EZA9) was reproducibly regulated by S1P without the addition of IL-1ß (Table S1). Figures S4–S6 show the biological processes, molecular functions, and cellular location of the 13 regulated proteins as categorized by the GO Slim categories (Table S2).

In the presence of IL-1ß, only 2 (P02795, P04733) of 8 proteins that were reproducibly and significantly regulated were stimulated by SPC, which is similar to the FLSs that were treated with IL-1ß alone but not reproducibly. Notably, SPC alone was ineffective. The biological processes, molecular functions, and cellular location of the eight proteins are shown in Figures S7–S9 (Table S2).

### 2.3. RT-PCR on Several Regulated Proteins

The mRNA expression was compared with the levels of some regulated proteins. For this purpose, 11 proteins that were reproducibly or significantly regulated by at least 1 treatment condition were determined using RT-PCR (Tables S1 and S2). A correlation analysis of 42 comparisons between the average AR and average fold-change in mRNA expression yielded a Spearman correlation coefficient of r = 0.68 (95% CI: 0.47 to 0.82; *p* < 0.0001).

### 2.4. Quantification of C1P and SPC in OA Knee Synovial Fluid

**Figure 6:**
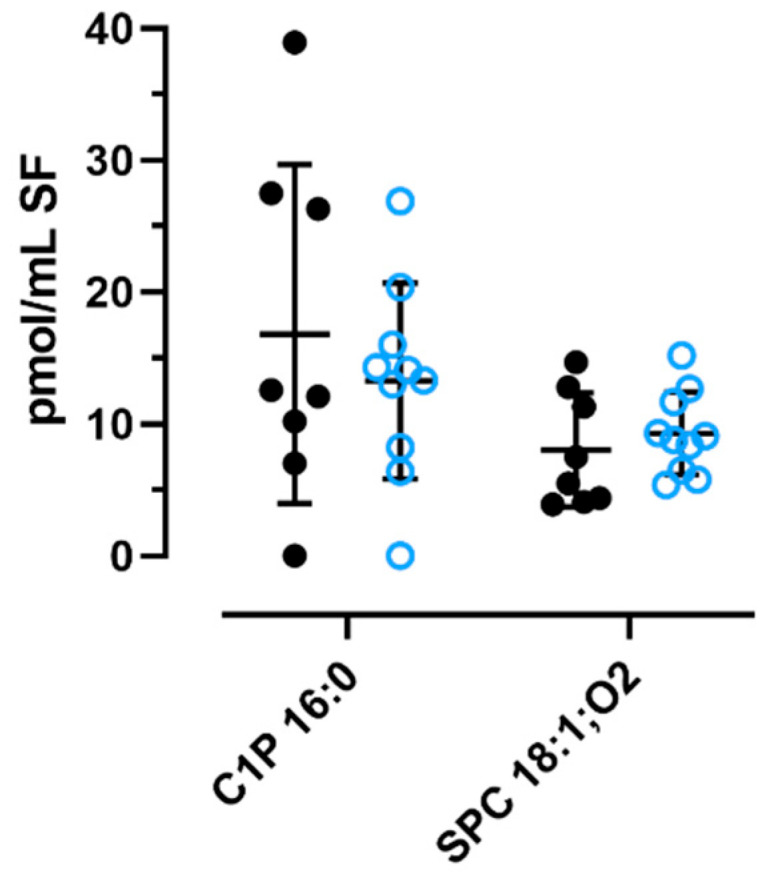
Levels of C1P 16:0 ad SPC 18:1;O2 in knee SF of patients with early-stage or late-stage knee OA. Sphingolipids were quantified by LC-MS/MS in extracts of SF obtained from 8 patients with eOA (black circle) and 10 patients with lOA (blue open circle). Data presented indicate mean ± SD of lipid concentration in SF (N = 8 or 10).

Next, we quantified the three sphingolipids in the SF of 8 eOA and 10 lOA patients using LC-MS/MS. We could not detect any S1P, because the values were below the limit of detection (6 pmol/mL). However, we reliably determined the concentrations of C1P 16:0 and SPC 18:1;O2 in patients with eOA (C1P: 16.8 ± 12.9 pmol/mL; SPC: 8.0 ± 4.3 pmol/mL) or lOA (C1P: 13.3 ± 7.4 pmol/mL; SPC 9.3 ± 3.1 pmol/mL). Also, as seen in Figure 6, the levels appear to be independent of disease stage. Unfortunately, none of the three sphingolipids could be detected in the SF of joint-healthy controls due to the dilutions that were performed in our laboratory.

## 3. Discussion

Sphingolipids have critical functions in structural integrity and cellular signaling in eukaryotic cells. However, our understanding of C1P, S1P, and SPC in articular joints is limited. Receptors for these sphingolipids have been identified on various cells, including chondrocytes, prompting an examination of their effects on articular cells, such as FLSs. Using novel MS technology, we quantified the impact of these sphingolipids on protein expression in FLSs. C1P emerged as the most active lipid, followed by S1P and SPC. Whereas C1P and S1P significantly regulated 9 proteins and 1 protein, respectively, SPC had no effect. Such variations in efficacy may be attributed to differential binding receptors, although their presence on FLSs requires confirmation [13,30,31].

A prior study on LPCs [9] confirmed the pleiotropic effects of IL-1ß on FLSs. Given our focus on sphingolipids, we abstained from re-evaluating the impact of IL-1ß on protein expression. However, its involvement in the biochemical pathways of various articular cells, including chondrocytes, synovial fibroblasts, osteoblasts, and osteoclasts, has been extensively documented [9,42,43]. Notably, IL-1ß has proinflammatory activity in the pathogenesis of OA, particularly its early stages [7,8]. Thus, we examined sphingolipid levels in the presence of IL-1ß, observing the significant and reproducible regulation of additional proteins, indicating lipid-specific effects on expression profiles.

In this study, we also quantified C1P, S1P, and SPC in the SF of 8 eOA and 10 lOA patients using LC-MS/MS. We could not detect any S1P, because its values were beneath the limit of detection (6 pmol/mL), whereas those of C1P 16:0 and SPC 18:1;O2 in all OA patients averaged 17.3 ± 14.4 pmol/mL and 8.6 ± 3.6 pmol/mL, respectively. Compared with their plasma levels in normo-lipidemic patients (C1P: 62 ± 20 pmol/mL, S1P: 530 ± 90 pmol/mL, SPC 18:1;O2: 53 ± 30 pmol/mL), the concentrations of sphingolipids in the SF are notably lower [12]. Despite adding 5% FBS to our cell culture media and assuming similar levels as in human serum, only minimal amounts of the three sphingolipids were added through FBS. Consequently, the applied lipid concentration of 1 nmol/mL for cultured FLSs far exceeds such concentrations in the SF, ensuring that the possible effects were maximized. However, additional studies are warranted to analyze the concentration-dependent effects that were observed.

Many proteins underwent consistent and significant stimulation (>1.2-fold) or inhibition (<0.8-fold) by the three sphingolipids. Each protein was analyzed twice by MS in 7 biological replicates, yielding data from 14 replicates per treatment. This meticulous approach was essential, because preliminary experiments indicated limited reproducibility between biological and technical replicates in MS data. Consequently, only those proteins exhibiting consistent directional regulation in at least 11 of the 14 replicates were viewed as being reproducible. This criterion ensured the reliability of our assessments of the effects of lipids.

Quantitative RT-PCR and WB were performed to analyze several proteins that exhibited significant and reproducible regulation. In our correlation analysis, there was a moderate correlation (r = 0.68) between the average ARs of select proteins and their corresponding mRNA levels. This observation aligns with previous findings that have indicated a limited correlation between mRNA and protein data due to technical and biological factors [44,45]. 

Our primary focus in this study was to identify proteins that were significantly regulated in OA with potential pathophysiological relevance. Among them was matrix metalloproteinase 3 (MMP-3; P08254), which has a well-established role in the pathophysiology of OA [46,47]. Notably, our findings demonstrated that only C1P significantly and reproducibly stimulated MMP-3 expression, albeit to a lesser extent than IL-1ß, a known regulator of MMP-3. MMP-3, also referred to as stromelysin-1, has a broad substrate specificity for extracellular matrix components, such as proteoglycans. It contributes to the progressive degradation of articular cartilage in OA [46]. Since 2003, researchers have postulated that maintaining a balance between MMPs and their natural tissue inhibitors of metalloproteinases (TIMPs) is critical to the integrity of the extracellular matrix [47]. In this context, our results suggest a potential catabolic effect of C1P based on the finding that it upregulates MMP-3; thus this delicate balance is potentially disrupted in the extracellular matrix.

C1P also has anti-catabolic effects, increasing the generation of superoxide dismutase (*SOD2*, Q7Z7M4), like IL-1ß. This enzyme (EC: 1.5.1.1) catalyzes the formation of hydrogen peroxide from superoxide anion radicals inside and outside of the cells; hydrogen peroxide is less harmful and is further degraded by reduction [48,49]. It is assumed that age-related mitochondrial dysfunction, accompanied by the reduced formation of mitochondrial superoxide dismutase, occurs, favoring the mitochondrial formation of reactive oxygen species in chondrocytes that damage articular cartilage [48,49].

This protective effect of C1P is also reflected in the presumed anti-apoptotic effect of charged multivesicular body protein 1b (*CHMP1B*, Q7LBR1), the expression of which is induced by C1P and IL-1ß. This protein (Q7LBR1) is associated with the endosomal sorting complex required for the transport-III (ESCRT-III) system, which is essential for mending injured plasma membranes [50]. 

Further, C1P upregulates cytosolic phospholipase A2 (*cPLA2*, P47712), which selectively hydrolyzes the sn-2 arachidonoyl group of membrane phospholipids, supplying eicosanoid biosynthesis precursors via the cyclooxygenase pathway [51]. Moreover, C1P is a strong and selective activator of this phospholipase, through interaction with the C2 domain [52]. The increased release of arachidonic acid and subsequent production of PGE2, also observed for IL-1ß [53], contribute to the inflammatory process in OA, suggesting a proinflammatory function for C1P in OA.

The upregulation of long-chain-fatty-acid–CoA ligase 4 (O60488) that is induced by C1P—more so by IL-1ß—is significant in this context. This enzyme catalyzes the conversion of long-chain fatty acids into their active acyl-CoA forms, vital for lipid synthesis and degradation. The polyunsaturated fatty acids arachidonate and eicosapentaenoate are its preferred substrates. Notably, this enzyme modulates the secretion of PGE2 in human arterial smooth muscle cells [54].

C1P upregulates other proteins that are involved in the inflammatory process and the progression of OA. Growth-regulated alpha protein, or CXCL1 (P09341), is stimulated by C1P, as well as by IL-1ß; this chemokine has a similar function as IL-8. After binding to its receptor CXCR2, CXCL1 activates multiple MAP kinases, and upregulates during and contributes to inflammation [55]. CXCL1 increases the expression of IL-6 in RA and OA synovial fibroblasts [56] and is upregulated in OA chondrocytes. Also, during cartilage development, CXCL1 promotes chondrocyte hypertrophy and triggers apoptosis [57,58].

The formation of the metal cation symporter ZIP14 (Q15043) and the intercellular adhesion molecule (ICAM-1) is stimulated by C1P and more strongly by IL-1ß. The metal cation symporter ZIP14 (Q15043) is a plasma membrane transporter that is involved in the uptake of divalent metal cations, such as zinc, manganese, and iron, and is crucial for tissue homeostasis, metabolism, development, and immunity. In particular, it shapes innate immunity by regulating cytokine expression in activated macrophages [59]. ICAM-1 (Q5NKV8), a glycoprotein of the immunoglobulin superfamily, binds to leukocyte β2 integrin and is implicated in the pathophysiology of OA, mediating inflammation and apoptosis and possibly facilitating crosstalk between bone and cartilage [60]. Whereas healthy synovium expresses low levels of ICAM-1, OA chondrocytes have a higher content [60]. 

C1P upregulated the enzyme glutamine-fructose-6-phosphate transaminase (*GFPT2*, A0A0S2Z4X9), which regulates the hexosamine biosynthetic pathway [61]. This transaminase catalyzes the conversion of D-fructose-6-phosphate and L-glutamine into D-glucosamine-6-phosphate, a precursor of glycosaminoglycans, components of proteoglycans in the extracellular matrix of, for example, articular cartilage [61]. The significance of alterations in the expression of this enzyme with regard to cartilage and joints remains to be determined, as does that of mitochondrial cytochrome c oxidase subunit 1 (Q4EZA9), which plays a key role in aerobic metabolism as an enzyme being stimulated only by S1P.

The two metallothioneins, MT-1F (P04733) and MT-2A (P02795), in addition to ferritin (A0A384MDR3, Q6NS36), were significantly modulated by C1P, S1P, and SPC in the presence of IL-1ß compared with IL-1ß alone. Originally linked to heavy metal detoxification, metallothioneins are now recognized for their involvement in various cellular processes, such as proliferation, migration, apoptosis, and antioxidation [62]. Elevated levels of MT-1 are associated with erosive inflammatory OA, wherein MT-1 has anti-inflammatory properties, inhibiting the expression of IL-1ß, TNF-alpha, and IL-6 in peripheral blood mononuclear cells and synovial cells in vitro [63]. MT-2 is implicated in inflammatory diseases of the respiratory and nervous systems and cancer [62], suggesting that the pharmacological induction of MT-2 could confer protection in inflammatory conditions. However, further research is needed to determine the (patho)physiological relevance of metallothioneins in joint health. 

The need for further investigations also extends to ferritin, a protein that is primarily involved in intracellular iron storage. Ferritin has a particular pathophysiological function as a suppressor of ferroptosis, a type of non-apoptotic cell death that depends on iron which has been known since 2012 [64]. The downregulation of ferritin by C1P, S1P, and SPC in the presence of IL-1ß by approximate 50% thus appears to promote ferroptosis in FLSs. Further, the functions of prosaposin (Q59EN5) and 40S ribosomal protein S27 (P42677) in the articular joint remain underexamined. For example, prosaposin, preferentially stimulated by S1P in the presence of IL-1ß compared with IL-ß alone, is a 65/70-kDa glycoprotein and a precursor of saposins, which subsequently activate enzymes responsible for degradation of specific sphingolipids [65]. Further studies are needed to determine the (patho)physiological functions of other proteins that are reproducibly and significantly regulated by C1P, S1P, or SPC in the presence of IL-1ß, such as thymosin (D6W5K2), dolichyl-diphosphooligosaccharide–protein glycosyltransferase (A0A6I8PLD9), RNA-binding motif protein 3 (A0A024QYX3), splicing factor (A8K644), 40S ribosomal protein S15 (P62841), thioredoxin-dependent peroxide reductase (P30048), GYG1 protein (Q8N5Y3), cDNA FLJ60294 (B4DLF7), and dynein light chain 2 (Q96FJ2). 

In conclusion, our study complements the poorly understood pathophysiologic role of the three major sphingolipids in the joints, of which C1P and S1P have emerged as the most active. Based on our findings, C1P-stimulated proteins with partially opposing effects as both pro- and anti-inflammatory and catabolic proteins were upregulated, in addition to other proteins. Our novel proteomic MS analysis yielded a large amount of data, showing that the expression profiles of proteins following treatment of FLSs with these sphingolipids change markedly when the proinflammatory cytokine IL-1ß is present. Thus, our in vitro model provides a more accurate representation of FLS responses across the various stages of OA, particularly with regard to the elevated IL-1ß levels that are observed in its early stages. Further, our study has identified several proteins that are implicated in the pathogenesis of OA throughout disease progression, although their specific functions require validation.

## 4. Materials and Methods

### 4.1. Materials 

Unless otherwise specified, all reagents were obtained from Sigma (Deisenhofen, Germany). Avanti Polar Lipids (Alabaster, AL, USA) provided Huzzah^®^ C1P [C16 ceramide-1-phosphate (18:1;O2/16:0), C1P 16:0], Huzzah^®^ S1P [sphingosine-1-phosphate (18:1;O2), S1P 18:1;O2] and sphingosylphosphorylcholine (SPC 18:1;O2, Lyso SM 18:1;O2). Aliquots of a stock solution of each lipid species were prepared according to the manufacturer’s instructions and stored frozen at −20 °C. After being thawed, the aliquots were mixed thoroughly and used to treat cultured FLSs as described below.

### 4.2. Specimen Selection for Isolation of FLSs 

Synovial tissue was donated by patients selected at random during knee replacement surgery at the Clinic and Polyclinic for Orthopedics and Orthopedic Surgery (Giessen), University Hospital Giessen and Marburg (Germany). All study procedures that involved patients were conducted per the Declaration of Helsinki. Approval was obtained from the Ethical Review Committee of the Faculty of Medicine of Justus Liebig University Giessen (Germany). All patients gave their written informed consent to donate samples for research and allow the publication of any resulting findings. The data presented in this paper do not allow for the identification of any individual patient. 

Donating patients had late-stage knee OA, had to be aged younger than 85 years, and had to have a BMI of <40 and a CRP level of ≤8 mg/dL. The impact of lipid species on the FLS proteome was not studied in cells from excluded patients, as detailed in references [4,5,9], such as those with other joint diseases, a serious illness such as tumor or HIV, or a severe disease of the kidney or liver. 

### 4.3. Culture of FLSs

Human FLSs were obtained from the synovial membranes of OA knee joints as described [66]. Briefly, FLSs were cultured up to passage 3–4 in DMEM (PAN Biotech, Aidenbach, Germany) with supplements under 10% CO2 at 37 °C. Mycoplasma contamination was ruled out using an I/C PCR mycoplasma test kit (PromoCell, Heidelberg, Germany). The purity of confluent FLSs was evaluated using FACS according to the previously outlined method [4,5] with 94.4% ± 5.8% showing positivity for CD90 and negativity for CD45.

### 4.4. Treatment of Cultured FLSs for Proteomic and mRNA Analysis 

The practical procedure for the treatment of cultured FLS for proteomic and mRNA analyses has already been published recently, whereby other lipid species were investigated [9]. Briefly, to analyze the FLS proteome, passage 4 FLSs were seeded in 6-well plates at 80,000 cells per well and grown to confluence in supplemented DMEM, changing the media every 2–3 days. Purity was verified by FACS. Cells were then treated with serum-reduced DMEM (5% FBS) containing folic acid for 24 h, followed by media with 1 µM C1P, S1P, or SPC, and/or 1 nM IL-1ß, whereas controls received vehicle only. After 48 h, the media was removed, cells were washed with PBS, and mycoplasma testing was performed. For mRNA analysis, cells were lysed in RP1 buffer with 1% β-mercaptoethanol following the manufacturer’s guideline (NucleoSpin^®^ RNA/Protein kit, Macherey-Nagel, Düren, Germany), subjected to sonication, snap frozen, and kept at −86 °C for storage. 

For the proteomic analysis, lipid-treated and untreated FLSs from the same biological replicates were washed with ice-cold PBS, lysed in 0.5 mL lysis buffer, sonicated, and kept at −86 °C for storage. 

This procedure was repeated 6 times with FLSs from 7 OA patients who underwent knee replacement surgery (age 68.3 ± 1.1 years, BMI 30.4 ± 4.2 kg/m^2^, CRP 3.1 ± 2.3 mg/mL, 3 males). Some patients had comorbidities, including arterial hypertension (*n* = 4), hypothyroidism (*n* = 3), hyperlipidemia (*n* = 2), and fibromyalgia (*n* = 1). Each of the 7 biological replicates was analyzed in duplicate, resulting in 14 proteomic data per treatment or control.

### 4.5. Isolation, TMT Labeling, and Mass Spectrometry of Cellular Proteins

The protein levels in lysed FLS extracts were measured and subjected to trypsin digestion as detailed earlier [9,67]. Briefly, tryptic peptides were desalted, lyophilized, redissolved in 50 mM HEPES, and quantified. 

For each of the 7 biological replicates, 10 µg of peptides from 8 treatments were labeled with TMT10plex reagents (Thermo Fisher Scientific, Waltham, MA, USA) according to the instructions of the manufacturer. Each isobaric reagent was assigned to a specific treatment or control as described below, using the pooled control sample as a reference for comparison: reporter ions at m/z = 126- untreated control, 127N-SPC, 127C-S1P, 128N-C1P, 128C-IL-1ß-treated control, 129N-SPC + IL-1ß, 129C-S1P + IL-1ß, 130N-C1P + IL-1β, and 130C-pool of the 7 untreated controls. 

MS analysis was performed as previously described [9,68]. Briefly, labeled peptide mixtures were fractionated into 8 fractions per replicate. For MS, 1 µg of each fraction was loaded onto a 50-cm µPAC™ C18 column (Pharma Fluidics, Gent, Belgium), eluted with a 240 min acetonitrile gradient, and analyzed with an Orbitrap Eclipse Tribrid mass spectrometer (Thermo Fisher Scientific, Waltham, MA, USA) in positive ionization mode.

### 4.6. Protein Identification and Quantitation

Proteins were identified and quantified as per established protocols [9,68]. Briefly, data acquisition was performed on an Xcalibur 4.3.73.11 (Thermo Fisher Scientific, Waltham, MA, USA). The merged data sets from 8 fractions per biological replicate were examined using Proteome Discoverer 2.5.0.400 (Thermo Fisher Scientific, Waltham, MA, USA). The search was performed with Sequest HT, a 10 ppm mass tolerance of precursor ions and 1 allowed missed cleavage. The FDR of identified peptides was limited to 0.01. Identified proteins required at least 1 unique peptide of 6–144 amino acids. 

### 4.7. Relative mRNA Expression 

RNA was isolated and measured with a 260/280 nm ratio of 1.98 ± 0.06 using a NanoDrop™ 2000 (Thermo Fisher Scientific, Waltham, MA, USA) as previously described [9]. Briefly, RNA was reverse-transcribed with the QuantiTect Reverse Transcription Kit (Qiagen, Hilden, Germany). The cDNA was then assessed in duplicate using QuantiNova LNA PCR or QuantiTECT Primer Assay (only MMP3) with the QuantiNova SYBR Green PCR kit (Qiagen) on a 7500 Fast Real-Time PCR system (Applied Biosystem, Waltham, MA, USA) for the following specified genes (Qiagen GeneGlobe ID): CXCL1 (SBH0404660), CHMP1B (SBH0502633), DYNLL2 (SBH0665947), FTH1 (SBH1220010), MMP3 (QT00060025), MT2A (SBH0576025), MT-CO1 (SBH0135562), PLA2G4A (SBH056400), PSAP (SBH0480826), and RPS27L (SBH0138199). The QuantiNova LNA PCR Assay and QuantiTECT Primer Assay (Qiagen) use predesigned, validated primer pairs specific to target cDNA. Primer efficiency [69] ranged from 94% to 103% (*n* = 12). The 2^−ΔΔCt^ method determined relative gene expression between treated and untreated FLSs, with GAPDH (SBH1220545) as the endogenous reference gene.

### 4.8. Sampling of Human Synovial Fluid

Human SF was obtained from 18 OA patients during arthroscopy (early-stage OA) or knee replacement surgery (late-stage OA) and prepared as described [1]. The Ethical Review Committee of the Faculty of Medicine (Justus Liebig University of Giessen, Germany) approved this study, and all patients provided written informed consent to donate samples for research. We used the Outerbridge classification scale (OU) to subcategorize changes in the joints during OA as early (eOA) and late OA (lOA), as defined earlier [1,2]. Eight patients with eOA (age 44.8 ± 13.1 years, BMI 27.4 ± 2.9 kg/m^2^, CRP 2.2 ± 2.1 mg/mL, 7 males) and ten patients with lOA (age 64.7 ± 13.0 years, BMI 31.0 ± 5.9 kg/m^2^, CRP 1.76 ± 1.0 mg/mL, 7 males) were included. Some patients had comorbidities, including cardiac insufficiency (lOA: *n* = 1), coronary heart disease (lOA: *n* = 1), hypertension (lOA: *n* = 9; eOA: *n* = 1), cardiac arrhythmia (lOA: *n* = 1), angina pectoris (lOA: *n* = 2), gout (lOA: *n* = 2), diabetes (eOA: *n* = 1), hay fever (lOA: *n* = 2; eOA: *n* = 1), and hypothyroidism (lOA: *n* = 2; eOA: *n* = 2). 

SF was obtained as previously described [1]. Briefly, fresh SF samples were checked for blood contamination and high turbidity, incubated for 15 min at 37 °C, and filtered to remove cells. Samples were then analyzed microscopically, treated with 10% protease and phospholipase inhibitors, centrifuged at 16,100× *g* for 45 min, and supernatant was frozen at −86 °C for subsequent analysis.

### 4.9. MS Analysis of Sphingolipids in Knee Synovial Fluid of OA Patients

Sphingolipids were extracted and quantified as detailed earlier [70,71]. In brief, lipids were extracted from the cell and cell debris-free supernatant of SF, and the concentrations of SPC [71], C1P [71], and S1P [70] were determined by liquid chromatographic separation MS/MS (LC-MS/MS). Lipid species were annotated as published [72].

### 4.10. Bioinformatics

We used Proteome Discoverer 2.5.0.400 to obtain protein annotations from the Gene Ontology (GO) database (https://geneontology.org/, accessed 22 February 2024) as previously described [9]. Briefly, for analyzing differentially expressed proteins, we retrieved information on biological processes, molecular functions, and cellular components. GO Slim terms from The Jackson Laboratory were modified with additional terms from the GO Enrichment Tool in Proteome Discoverer to enrich the proteins with OA-relevant information.

### 4.11. Statistical Analysis 

The data analysis and charts were generated using GraphPad Prism version 9.5.1 for Windows (GraphPad Software, San Diego, CA, USA) and Proteome Discoverer version 2.5.0.400 (Thermo Fisher Scientific, Waltham, MA, USA). Venn diagrams were drawn using a web-based tool [73]. The proteins used for the proteomic analysis were sourced from cell extracts of both treated and untreated FLS cultures, all from the same patient. The experiments were repeated 6 times, using samples from 7 OA patients; thus, 7 biological replicates per treatment were analyzed, with each replicate being assessed in duplicate (*n* = 7). In total, proteomic data were collected from 14 replicates for each treated or untreated FLS culture.

To address skewness, both the abundance and abundance ratios (ARs) were log-transformed before including them in the analysis. The subsequent analysis adhered to the following 3 prerequisites: (1) ARs of proteins that were reproducibly regulated by C1P, S1P, SPC, IL-1ß, or IL-1ß in the presence of C1P, S1P, or SPC are above 1.2-fold or below 0.8-fold relative to untreated controls in at least 11 of 14 replicates. (2) On average, ARs of proteins from treated FLSs had to be over 1.2-fold or below 0.8-fold relative to untreated controls. (3) ARs of proteins from FLSs treated with C1P, S1P, or SPC in the presence of IL-1ß had to be above 1.2-fold or below 0.8-fold relative to FLSs that were administered IL-1ß alone in at least 11 of 14 replicates. 

The adjusted *p*-values for comparing the levels between treated and untreated controls (Table S1) were calculated using repeated-measures one-way ANOVA, followed by Dunnett’s multiple comparisons test. Adjusted *p*-values for comparing 2 ARs (Table S2) were determined through multiple paired *t*-tests, with *p*-values corrected for multiple comparisons using the Holm–Sidak method. 

ARs are reported as mean ± SD, and those present in at least 11 of the 14 consistently regulated replicates are highlighted in bold (Tables S1 and S2). The significance threshold was set at *p* ≤ 0.05. Additionally, a Spearman correlation analysis was conducted to assess the relationship between the mean AR of selected proteins and the mean fold-change in their respective mRNA levels.

## Data Availability

The data in this study are available on reasonable request to the corresponding author.

## Supplementary Materials

The following supporting information can be downloaded at: https://www.mdpi.com/article/10.3390/ijms25158363/s1.

## Abbreviations

AR, abundance ratio; C1P, ceramide-1-phosphate; FDR, false discovery rate; FLSs, fibroblast-like synoviocytes; GO, gene ontology; IGF-I, insulin-like growth factor-1; IL-1ß, interleukin-1ß; KL score, Kellgren–Lawrence score; LPC, lysophosphatidylcholine; OA, osteoarthritis; PC, phosphatidylcholine; PL, phospholipids; RA, rheumatoid arthritis; RT-PCR, reverse-transcription polymerase chain reaction; S1P, sphingosine-1-phosphate; SM, sphingomyelin; SF, synovial fluid; SPC, sphingosylphosphorylcholine; TMT, tandem mass tag; TGF-ß1, transforming growth factor-ß1.

## References

[1] Kosinska M.K., Liebisch G., Lochnit G., Wilhelm J., Klein H., Kaesser U., Lasczkowski G., Rickert M., Schmitz G., Steinmeyer J. (2013). A lipidomic study of phospholipid classes and species in human synovial fluid. Arthritis Rheum..

[2] Kosinska M.K., Liebisch G., Lochnit G., Wilhelm J., Klein H., Kaesser U., Lasczkowski G., Rickert M., Schmitz G., Steinmeyer J. (2014). Sphingolipids in human synovial fluid—A lipidomic study. PLoS ONE.

[3] Kosinska M.K., Ludwig T.E., Liebisch G., Zhang R., Siebert H.-C., Wilhelm J., Kaesser U., Dettmeyer R.B., Klein H., Ishaque B. (2015). Articular Joint Lubricants during Osteoarthritis and Rheumatoid Arthritis Display Altered Levels and Molecular Species. PLoS ONE.

[4] Thottakkattumana Parameswaran V., Hild C., Eichner G., Ishaque B., Rickert M., Steinmeyer J. (2022). Interleukin-1 Induces the Release of Lubricating Phospholipids from Human Osteoarthritic Fibroblast-like Synoviocytes. Int. J. Mol. Sci..

[5] Sluzalska K.D., Liebisch G., Wilhelm J., Ishaque B., Hackstein H., Schmitz G., Rickert M., Steinmeyer J. (2017). Growth factors regulate phospholipid biosynthesis in human fibroblast-like synoviocytes obtained from osteoarthritic knees. Sci. Rep..

[6] Sluzalska K.D., Liebisch G., Lochnit G., Ishaque B., Hackstein H., Schmitz G., Rickert M., Steinmeyer J. (2017). Interleukin-1β affects the phospholipid biosynthesis of fibroblast-like synoviocytes from human osteoarthritic knee joints. Osteoarthr. Cartil..

[7] Ingale D., Kulkarni P., Electricwala A., Moghe A., Kamyab S., Jagtap S., Martson A., Koks S., Harsulkar A. (2021). Synovium-Synovial Fluid Axis in Osteoarthritis Pathology: A Key Regulator of the Cartilage Degradation Process. Genes.

[8] Ning L., Ishijima M., Kaneko H., Kurihara H., Arikawa-Hirasawa E., Kubota M., Liu L., Xu Z., Futami I., Yusup A. (2011). Correlations between both the expression levels of inflammatory mediators and growth factor in medial perimeniscal synovial tissue and the severity of medial knee osteoarthritis. Int. Orthop..

[9] Timm T., Hild C., Liebisch G., Rickert M., Lochnit G., Steinmeyer J. (2023). Functional Characterization of Lysophospholipids by Proteomic and Lipidomic Analysis of Fibroblast-like Synoviocytes. Cells.

[10] Christie W.W. Ceramide-1-Phosphate. The LipidWeb. https://www.lipidmaps.org/resources/lipidweb/index.php?page=lipids/sphingo/cer-1-p/index.htm.

[11] Graf C., Zemann B., Rovina P., Urtz N., Schanzer A., Reuschel R., Mechtcheriakova D., Müller M., Fischer E., Reichel C. (2008). Neutropenia with impaired immune response to Streptococcus pneumoniae in ceramide kinase-deficient mice. J. Immunol..

[12] Scherer M., Böttcher A., Schmitz G., Liebisch G. (2011). Sphingolipid profiling of human plasma and FPLC-separated lipoprotein fractions by hydrophilic interaction chromatography tandem mass spectrometry. Biochim. Biophys. Acta.

[13] Granado M.H., Gangoiti P., Ouro A., Arana L., González M., Trueba M., Gómez-Muñoz A. (2009). Ceramide 1-phosphate (C1P) promotes cell migration Involvement of a specific C1P receptor. Cell. Signal..

[14] Presa N., Gomez-Larrauri A., Rivera I.-G., Ordoñez M., Trueba M., Gomez-Muñoz A. (2016). Regulation of cell migration and inflammation by ceramide 1-phosphate. Biochim. Biophys. Acta.

[15] Gomez-Muñoz A., Presa N., Gomez-Larrauri A., Rivera I.-G., Trueba M., Ordoñez M. (2016). Control of inflammatory responses by ceramide, sphingosine 1-phosphate and ceramide 1-phosphate. Prog. Lipid Res..

[16] Gomez-Larrauri A., Presa N., Dominguez-Herrera A., Ouro A., Trueba M., Gomez-Muñoz A. (2020). Role of bioactive sphingolipids in physiology and pathology. Essays Biochem..

[17] Grösch S., Alessenko A.V., Albi E. (2018). The Many Facets of Sphingolipids in the Specific Phases of Acute Inflammatory Response. Mediat. Inflamm..

[18] Pettus B.J., Bielawska A., Subramanian P., Wijesinghe D.S., Maceyka M., Leslie C.C., Evans J.H., Freiberg J., Roddy P., Hannun Y.A. (2004). Ceramide 1-phosphate is a direct activator of cytosolic phospholipase A2. J. Biol. Chem..

[19] Lamour N.F., Chalfant C.E. (2008). Ceramide kinase and the ceramide-1-phosphate/cPLA2alpha interaction as a therapeutic target. Curr. Drug Targets.

[20] Lamour N.F., Subramanian P., Wijesinghe D.S., Stahelin R.V., Bonventre J.V., Chalfant C.E. (2009). Ceramide 1-phosphate is required for the translocation of group IVA cytosolic phospholipase A2 and prostaglandin synthesis. J. Biol. Chem..

[21] Hankins J.L., Fox T.E., Barth B.M., Unrath K.A., Kester M. (2011). Exogenous ceramide-1-phosphate reduces lipopolysaccharide (LPS)-mediated cytokine expression. J. Biol. Chem..

[22] Christie W.W. Sphingosine-1-Phosphate. The LipidWeb. https://www.lipidmaps.org/resources/lipidweb/index.php?page=lipids/sphingo/sph-1-p/index.htm.

[23] Kitano M., Hla T., Sekiguchi M., Kawahito Y., Yoshimura R., Miyazawa K., Iwasaki T., Sano H., Saba J.D., Tam Y.Y. (2006). Sphingosine 1-phosphate/sphingosine 1-phosphate receptor 1 signaling in rheumatoid synovium: Regulation of synovial proliferation and inflammatory gene expression. Arthritis Rheum..

[24] Lucaciu A., Brunkhorst R., Pfeilschifter J.M., Pfeilschifter W., Subburayalu J. (2020). The S1P–S1PR Axis in Neurological Disorders—Insights into Current and Future Therapeutic Perspectives. Cells.

[25] Weske S. (2018). Targeting sphingosine-1-phosphate lyase as an anabolic therapy for bone loss. Nat. Med..

[26] Moritz E., Wegner D., Groß S., Bahls M., Dörr M., Felix S.B., Ittermann T., Oswald S., Nauck M., Friedrich N. (2017). Reference intervals for serum sphingosine-1-phosphate in the population-based Study of Health in Pomerania. Clin. Chim. Acta.

[27] Moritz E., Jedlitschky G., Negnal J., Tzvetkov M.V., Daum G., Dörr M., Felix S.B., Völzke H., Nauck M., Schwedhelm E. (2021). Increased Sphingosine-1-Phosphate Serum Concentrations in Subjects with Periodontitis: A Matter of Inflammation. J. Inflamm. Res..

[28] Fitzpatrick L.R., Green C., Maines L.W., Smith C.D. (2011). Experimental osteoarthritis in rats is attenuated by ABC294640, a selective inhibitor of sphingosine kinase-2. Pharmacology.

[29] El Jamal A., Bougault C., Mebarek S., Magne D., Cuvillier O., Brizuela L. (2020). The role of sphingosine 1-phosphate metabolism in bone and joint pathologies and ectopic calcification. Bone.

[30] Masuko K., Murata M., Beppu M., Nakamura H., Kato T., Yudoh K. (2012). Sphingosine-1-phosphate modulates expression of vascular endothelial growth factor in human articular chondrocytes: A possible new role in arthritis. Int. J. Rheum. Dis..

[31] Masuko K., Murata M., Nakamura H., Yudoh K., Nishioka K., Kato T. (2007). Sphingosine-1-phosphate attenuates proteoglycan aggrecan expression via production of prostaglandin E2 from human articular chondrocytes. BMC Musculoskelet. Disord..

[32] Stradner M.H., Gruber G., Angerer H., Huber V., Setznagl D., Kremser M.-L., Moazedi-Fürst F.C., Windhager R., Graninger W.B. (2013). Sphingosine 1-phosphate counteracts the effects of interleukin-1β in human chondrocytes. Arthritis Rheum..

[33] Cherifi C., Latourte A., Vettorazzi S., Tuckermann J., Provot S., Ea H.-K., Ledoux A., Casas J., Cuvillier O., Richette P. (2021). Inhibition of sphingosine 1-phosphate protects mice against chondrocyte catabolism and osteoarthritis. Osteoarthr. Cartil..

[34] Kim M.-K., Lee H.Y., Kwak J.-Y., Park J.-I., Yun J., Bae Y.-S. (2006). Sphingosine-1-phosphate stimulates rat primary chondrocyte proliferation. Biochem. Biophys. Res. Commun..

[35] Meyer zu Heringdorf D., Himmel H.M., Jakobs K.H. (2002). Sphingosylphosphorylcholine-biological functions and mechanisms of action. Biochim. Biophys. Acta.

[36] Wirrig C., Hunter I., Mathieson F.A., Nixon G.F. (2011). Sphingosylphosphorylcholine is a proinflammatory mediator in cerebral arteries. J. Cereb. Blood Flow Metab..

[37] Christie W.W. Sphingomyelin and Related Sphingophospholipids. https://www.lipidmaps.org/resources/lipidweb/index.php?page=lipids/sphingo/sph/index.htm.

[38] Park M.K., Lee C.H. (2019). Role of Sphingosylphosphorylcholine in Tumor and Tumor Microenvironment. Cancers.

[39] Liliom K., Sun G., Bünemann M., Virág T., Nusser N., Baker D.L., Wang D.A., Fabian M.J., Brandts B., Bender K. (2001). Sphingosylphosphocholine is a naturally occurring lipid mediator in blood plasma: A possible role in regulating cardiac function via sphingolipid receptors. Biochem. J..

[40] Maglaviceanu A., Wu B., Kapoor M. (2021). Fibroblast-like synoviocytes: Role in synovial fibrosis associated with osteoarthritis. Wound Repair Regen..

[41] Han D., Fang Y., Tan X., Jiang H., Gong X., Wang X., Hong W., Tu J., Wei W. (2020). The emerging role of fibroblast-like synoviocytes-mediated synovitis in osteoarthritis: An update. J. Cell. Mol. Med..

[42] Jenei-Lanzl Z., Meurer A., Zaucke F. (2019). Interleukin-1β signaling in osteoarthritis—Chondrocytes in focus. Cell. Signal..

[43] Shen S., Guo J., Luo Y., Zhang W., Cui Y., Wang Q., Zhang Z., Wang T. (2014). Functional proteomics revealed IL-1β amplifies TNF downstream protein signals in human synoviocytes in a TNF-independent manner. Biochem. Biophys. Res. Commun..

[44] Buccitelli C., Selbach M. (2020). mRNAs, proteins and the emerging principles of gene expression control. Nat. Rev. Genet..

[45] Schwanhäusser B., Busse D., Li N., Dittmar G., Schuchhardt J., Wolf J., Chen W., Selbach M. (2011). Global quantification of mammalian gene expression control. Nature.

[46] Mukherjee A., Das B. (2024). The role of inflammatory mediators and matrix metalloproteinases (MMPs) in the progression of osteoarthritis. Biomater. Biosyst..

[47] Wan J., Zhang G., Li X., Qiu X., Ouyang J., Dai J., Min S. (2021). Matrix Metalloproteinase 3: A Promoting and Destabilizing Factor in the Pathogenesis of Disease and Cell Differentiation. Front. Physiol..

[48] Bolduc J.A., Collins J.A., Loeser R.F. (2019). Reactive oxygen species, aging and articular cartilage homeostasis. Free Radic. Biol. Med..

[49] Liu L., Luo P., Yang M., Wang J., Hou W., Xu P. (2022). The role of oxidative stress in the development of knee osteoarthritis: A comprehensive research review. Front. Mol. Biosci..

[50] Yang Y., Wang M., Zhang Y.-Y., Zhao S.-Z., Gu S. (2022). The endosomal sorting complex required for transport repairs the membrane to delay cell death. Front. Oncol..

[51] Adler D.H., Cogan J.D., Phillips J.A., Schnetz-Boutaud N., Milne G.L., Iverson T., Stein J.A., Brenner D.A., Morrow J.D., Boutaud O. (2008). Inherited human cPLA(2alpha) deficiency is associated with impaired eicosanoid biosynthesis, small intestinal ulceration, and platelet dysfunction. J. Clin. Investig..

[52] Stahelin R.V., Subramanian P., Vora M., Cho W., Chalfant C.E. (2007). Ceramide-1-phosphate binds group IVA cytosolic phospholipase a2 via a novel site in the C2 domain. J. Biol. Chem..

[53] Leistad L., Feuerherm A.J., Faxvaag A., Johansen B. (2011). Multiple phospholipase A2 enzymes participate in the inflammatory process in osteoarthritic cartilage. Scand. J. Rheumatol..

[54] Golej D.L., Askari B., Kramer F., Barnhart S., Vivekanandan-Giri A., Pennathur S., Bornfeldt K.E. (2011). Long-chain acyl-CoA synthetase 4 modulates prostaglandin E₂ release from human arterial smooth muscle cells. J. Lipid Res..

[55] Silva R.L., Lopes A.H., Guimarães R.M., Cunha T.M. (2017). CXCL1/CXCR2 signaling in pathological pain: Role in peripheral and central sensitization. Neurobiol. Dis..

[56] Hou S.-M., Chen P.-C., Lin C.-M., Fang M.-L., Chi M.-C., Liu J.-F. (2020). CXCL1 contributes to IL-6 expression in osteoarthritis and rheumatoid arthritis synovial fibroblasts by CXCR2, c-Raf, MAPK, and AP-1 pathway. Arthritis Res. Ther..

[57] Merz D., Liu R., Johnson K., Terkeltaub R. (2003). IL-8/CXCL8 and growth-related oncogene alpha/CXCL1 induce chondrocyte hypertrophic differentiation. J. Immunol..

[58] Olivotto E., Vitellozzi R., Fernandez P., Falcieri E., Battistelli M., Burattini S., Facchini A., Flamigni F., Santi S., Facchini A. (2007). Chondrocyte hypertrophy and apoptosis induced by GROalpha require three-dimensional interaction with the extracellular matrix and a co-receptor role of chondroitin sulfate and are associated with the mitochondrial splicing variant of cathepsin B. J. Cell. Physiol..

[59] Sayadi A., Nguyen A.-T., Bard F.A., Bard-Chapeau E.A. (2013). Zip14 expression induced by lipopolysaccharides in macrophages attenuates inflammatory response. Inflamm. Res..

[60] Lavigne P., Benderdour M., Shi Q., Lajeunesse D., Fernandes J.C. (2005). Involvement of ICAM-1 in bone metabolism: A potential target in the treatment of bone diseases?. Expert Opin. Biol. Ther..

[61] Kroef V., Ruegenberg S., Horn M., Allmeroth K., Ebert L., Bozkus S., Miethe S., Elling U., Schermer B., Baumann U. (2022). GFPT2/GFAT2 and AMDHD2 act in tandem to control the hexosamine pathway. Elife.

[62] Wang X.-L., Schnoor M., Yin L.-M. (2023). Metallothionein-2: An emerging target in inflammatory diseases and cancers. Pharmacol. Ther..

[63] Dai H., Wang L., Li L., Huang Z., Ye L. (2021). Metallothionein 1: A New Spotlight on Inflammatory Diseases. Front. Immunol..

[64] Tang D., Chen X., Kang R., Kroemer G. (2021). Ferroptosis: Molecular mechanisms and health implications. Cell Res..

[65] Kishimoto Y., Hiraiwa M., O’Brien J.S. (1992). Saposins: Structure, function, distribution, and molecular genetics. J. Lipid Res..

[66] Neumann E., Riepl B., Knedla A., Lefèvre S., Tarner I.H., Grifka J., Steinmeyer J., Schölmerich J., Gay S., Müller-Ladner U. (2010). Cell culture and passaging alters gene expression pattern and proliferation rate in rheumatoid arthritis synovial fibroblasts. Arthritis Res. Ther..

[67] Wiśniewski J.R., Zougman A., Nagaraj N., Mann M. (2009). Universal sample preparation method for proteome analysis. Nat. Methods.

[68] Maheshwari G., Wen G., Gessner D.K., Ringseis R., Lochnit G., Eder K., Zorn H., Timm T. (2021). Tandem mass tag-based proteomics for studying the effects of a biotechnologically produced oyster mushroom against hepatic steatosis in obese Zucker rats. J. Proteom..

[69] Schmittgen T.D., Livak K.J. (2008). Analyzing real-time PCR data by the comparative C(T) method. Nat. Protoc..

[70] Scherer M., Schmitz G., Liebisch G. (2009). High-throughput analysis of sphingosine 1-phosphate, sphinganine 1-phosphate, and lysophosphatidic acid in plasma samples by liquid chromatography-tandem mass spectrometry. Clin. Chem..

[71] Scherer M., Leuthäuser-Jaschinski K., Ecker J., Schmitz G., Liebisch G. (2010). A rapid and quantitative LC-MS/MS method to profile sphingolipids. J. Lipid Res..

[72] Liebisch G., Fahy E., Aoki J., Dennis E.A., Durand T., Ejsing C.S., Fedorova M., Feussner I., Griffiths W.J., Köfeler H. (2020). Update on LIPID MAPS classification, nomenclature, and shorthand notation for MS-derived lipid structures. J. Lipid Res..

[73] Heberle H., Meirelles G.V., Da Silva F.R., Telles G.P., Minghim R. (2015). InteractiVenn: A web-based tool for the analysis of sets through Venn diagrams. BMC Bioinform..

