# Supplementary material for: Functional Insights into the Sphingolipids C1P, S1P, and SPC in Human Fibroblast-like Synoviocytes by Proteomic Analysis
Source: Int J Mol Sci. 2024 Jul 31;25(15):8363. doi: 10.3390/ijms25158363 (PMC11313292; doi:10.3390/ijms25158363)
Supplement: Supplementary file 1 [file ijms-25-08363-s001.zip › ijms-3111674-supplementary.pdf]

Supplementary Information

Functional Insights into the Sphingolipids C1P, S1P, and SPC in Human Fibroblast-Like Synoviocytes by Proteomic Analysis

Thomas Timm [1], Christiane Hild [2], Gerhard Liebisch [3], Markus Rickert [2], Guenter Lochnit [1] and Juergen Steinmeyer [2,*]

[1] Protein Analytics Group, Institute of Biochemistry, Justus Liebig University Giessen, 35392 Giessen, Germany
[2] Laboratory for Experimental Orthopedics, Department of Orthopedics, Justus Liebig University Giessen, 35392 Giessen, Germany
[3] Department for Clinical Chemistry and Laboratory Medicine, University Hospital Regensburg, 93053 Regensburg, Germany
* Correspondence: just-OA@online.de

Table S1. Proteins reproducibly regulated by C1P, S1P and SPC in human FLSs.

Accession ID	Gene	Protein name	AR C1P	AR S1P	AR SPC	AR IL-1ß
**P09341**	*CXCL1*	Growth-regulated alpha protein	**22.8 ± 38.8** * *(1.34±0.70)*	22.3 ± 38.9 *(1.54±1.20)*	15.1 ± 37.4 *(1.52±0.92)*	**17.3 ± 15.5** ** *(482±329)*
**P08254**	*MMP3*	Stromelysin-1	**1.70 ± 0.68** *** *(0.75±0.37)*	1.14 ± 0.48 *(1.21±0.87)*	1.01 ± 0.45 *(1.36±0.85)*	**15.2 ± 9.65** **
**Q7LBR1**	*CHMP1B*	Charged multivesicular body protein 1b	**1.49 ± 0.39** * *(1.23±0.50)*	1.45 ± 0.44 *(1.49±0.77)*	1.23 ± 0.27 *(1.19±0.48)*	**5.01 ± 2.31** **
**Q15043**	*SLC39A14*	Metal cation symporter ZIP14	**1.45 ± 0.27** ** *(0.88±0.25)*	1.16 ± 0.17 *(0.98±0.53)*	1.00 ± 0.13 *(1.29±0.47)*	**7.73 ± 3.16** **
**P47712**	*PLA2G4A*	Cytosolic phospholipase A2	**1.32 ± 0.11** *** *(0.89±0.16)*	1.20 ± 0.09 ** *(0.84±0.35)*	1.08 ± 0.08 *(1.08±0.32)*	**3.43 ± 1.47** **
**Q4EZA9**	*MT-CO1*	Cytochrome c oxidase subunit 1	1.32 ± 0.39 *(1.03±0.24)*	**1.58 ± 0.33** ** *(1.13±0.32)*	1.39 ± 0.34 * *(0.99±0.20)*	1.05 ± 0.28
**Q7Z7M4**	*SOD2*	Superoxide dismutase (Fragment)	**1.30 ± 0.20** *	1.23 ± 0.17 *	1.14 ± 0.16	**4.35 ± 1.63** **
**O60488**	*ACSL4*	Long-chain-fatty-acid--CoA ligase 4	**1.29 ± 0.18** *	1.15 ± 0.09 *	1.04 ± 0.11	**4.76 ± 1.36** ***
**Q5NKV8**	*ICAM1*	Intercellular adhesion molecule 1	**1.29 ± 0.17** **	1.17 ± 0.12 *	1.01 ± 0.03	**4.17 ± 1.34** **
**A0A0S2Z4X9**	*GFPT2*	Glutamine-fructose-6-P transaminase (isomerizing)	**1.26 ± 0.05** ***	1.15 ± 0.10 *	1.05 ± 0.07	**3.94 ± 1.34** **

Proteins are ordered according to their mean abundance ratio (AR) in C1P-treated FLSs. The ARs of protein levels in FLSs treated with S1P, SPC and IL-1β are shown relative to the abundance of their untreated controls. Accession IDs, gene symbols, and protein names were obtained from UniProt Knowledgebase. The ARs are presented as mean ± SD, and those reproducibly regulated in at least 11 of 14 replicates are shown in bold. The mRNA expression of select proteins was determined by RT-PCR using the $2^{-\Delta\Delta Ct}$ method. The data are presented in brackets and cursive style as mean ± SD of the fold-change in mRNA expression relative to untreated control (normalized to 1). *0.05 ≥ *P* > 0.01; **0.01 ≥ *P* >0.001; ****P* ≤ 0.001.

Table S2. The 19 proteins reproducibly regulated by C1P, S1P and/or SPC in the presence of IL-1ß.

Accession ID	Gene	Protein name	AR C1P+IL-1ß	AR S1P+IL-1ß	AR SPC+IL-1ß	AR IL-1ß
**P09341**	*CXCL1*	Growth-regulated alpha protein	20.8 ± 18.2	22.5 ± 20.9<br>*	**22.7 ± 21.8**	17.3 ± 15.5
			(503±256)	*(449±275)*	*(471±321)*	*(482±329)*
**P02795**	*MT2A*	Metallothionein-2A	**7.13 ± 2.55**<br>**	**7.09 ± 3.15**<br>**	**6.38 ± 3.24**<br>*	3.47 ± 1.47
			(4.34±2.42)	*(4.19±2.45)*	*(4.06±1.92)*	*(4.44±1.99)*
**P04733**	*MT1F*	Metallothionein-1F	**6.23 ± 3.05**<br>*	**6.76 ± 4.92**<br>*	**6.23 ± 4.61**<br>*	3.89 ± 2.91
**P09038-1**	*FGF2*	Isoform 2 of Fibroblast growth factor 2	1.84 ± 0.46	**1.95 ± 0.46**<br>**	1.70 ± 0.35	1.49 ± 0.32
**D6W5K2**	*TMSB10*	Thymosin, ß 10, isoform CRA_a (Fragment)	1.59 ± 0.77	**1.71 ± 0.76**<br>**	1.64 ± 0.52<br>*	1.31 ± 0.46
**Q59EN5**	*PSAP*	Prosaposin	1.59 ± 1.27	**1.80 ± 1.44**<br>**	1.51 ± 1.11	1.21 ± 1.07
			(0.96±0.36)	*(0.98±0.28)*	*(0.83±0.22)*	*(0.94±0.24)*
**A0A6I8PLD9**	*KRTCAP2*	Dolichyl-diphosphooligosaccharide--protein glycosyltransferase subunit KCP2	**1.43 ± 0.40**<br>**	**1.41 ± 0.38**<br>**	**1.41 ± 0.41**<br>*	1.02 ± 0.21
**O60476**	*MAN1A2*	Mannosyl-oligosaccharide 1,2-α-mannosidase IB	1.38 ± 0.30	**6.15 ± 3.33**<br>*	1.76 ± 0.48	1.50 ± 0.23
**P42677**	*RPS27*	40S ribosomal protein S27	**1.33 ± 0.26**<br>***	1.23 ± 0.28<br>***	1.13 ± 0.24	1.05 ± 0.24
**Q71UM5**	*RPS27L*	40S ribosomal protein S27-like	**1.19 ± 0.19**<br>**	0.87 ± 0.12<br>**	0.77 ± 0.11	0.78 ± 0.12
			(0.47±0.06)	*(0.47±0.05)*	*(0.52±0.08)*	*(0.50±0.08)*
**A0A024QYX3**	*RBM3*	RNA binding motif (RNP1, RRM) protein 3, isoform CRA_c	**1.19 ± 0.23**<br>*	1.18 ± 0.21<br>*	**1.17 ± 0.17**<br>*	0.87 ± 0.25
**A8K644**	*SFRS4*	Splicing factor, arginine/serine-rich 4, isoform CRA_b	**1.18 ± 0.22**	1.16 ± 0.17<br>*	1.15 ± 0.13<br>*	0.90 ± 0.17

**P62841**	*RPS15*	40S ribosomal protein S15	**1.13 ± 0.25** **	1.13 ± 0.28 **	1.10 ± 0.22 *	0.90 ± 0.25
**P30048**	*PRDX3*	Thioredoxin-dependent per-oxide reductase, mitochondrial	**1.08 ± 0.12**	**1.11 ± 0.16** *	**1.12 ± 0.17** *	0.89 ± 0.24
**Q8N5Y3**	*GYG1*	GYG1 protein (Fragment)	0.89 ± 0.25	**0.83 ± 0.20** **	0.86 ± 0.17 *	1.17 ± 0.23
**B4DLF7**		cDNA FLJ60294	**0.81 ± 0.20** *	**0.80 ± 0.18** **	**0.83 ± 0.18** *	2.95 ± 1.40
**Q96FJ2**	*DYNLL2*	Dynein light chain 2, cytoplasmic	**0.68 ± 0.43**	**0.66 ± 0.44** *	**0.61 ± 0.41** *	1.04 ± 0.76
			(0.70±0.29)	*(0.88±0.24)*	*0.77±0.28)*	*(0.84±0.27)*
**A0A384MDR3**	*FTL*	Ferritin	**0.58 ± 0.18** **	**0.58 ± 0.22** *	**0.59 ± 0.26**	0.80 ± 0.19
**Q6NS36**	*FTH1*	Ferritin (Fragment)	**0.57 ± 0.16** *	**0.56 ± 0.15** **	**0.58 ± 0.18** *	0.91 ± 0.15
			(0.93±0.13)	*(0.93±0.09)*	*0.93±0.10)*	*(1.05±0.18)*

Proteins are ordered according to their mean abundance ratio (AR) in FLS treated with C1P in the presence of IL-1ß. The ARs of protein levels in human FLSs treated with C1P, S1P or SPC in the presence of IL-1ß and IL-1β alone are shown relative to the abundance of their untreated controls. Accession IDs, gene symbols, and protein names were obtained from UniProt Knowledgebase. The ARs are presented as mean ± SD, and those reproducibly regulated above 1.2-fold or below 0.8-fold relative to FLS treated with IL-1ß alone in at least 11 of 14 replicates are shown in bold. The mRNA expression of select proteins was determined by RT-PCR using the $2^{-\Delta\Delta Ct}$ method. The data are presented in brackets and cursive style as mean ± SD of the fold-change in mRNA expression relative to untreated control (normalized to 1). Significantly different to the AR IL-1ß as indicated by the Holm-Sidak-adjusted P-value: *0.05 ≥ *P* > 0.01; **0.01 ≥ *P* >0.001; ****P* ≤ 0.001.

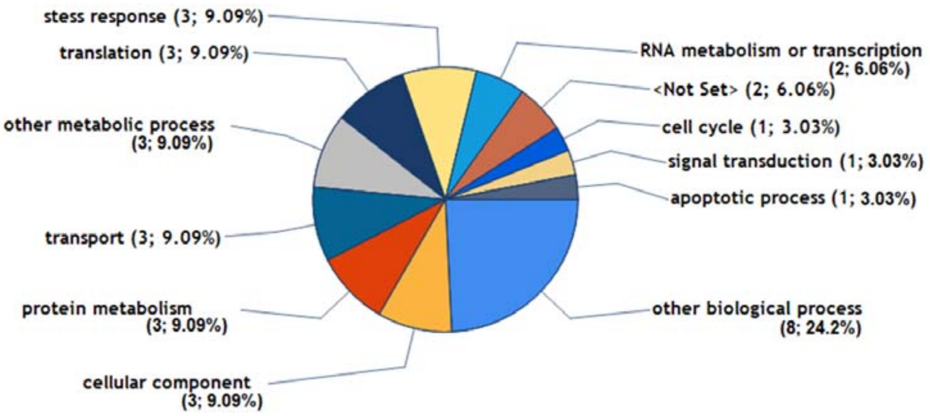

Figure S1. The biological processes of FLS being altered by C1P in the presence of IL-1ß. The 10 proteins were reproducibly upregulated by at least 1.2-fold or downregulated by 0.8-fold in FLS by C1P + IL-1ß during 48 h of treatment relative to FLS treated with IL-1ß alone. Table S2 provides further data on these proteins. The Go Slim categories for proteins were generated by Proteome Discoverer 2.5 software using the Gene Ontology (GO) database.

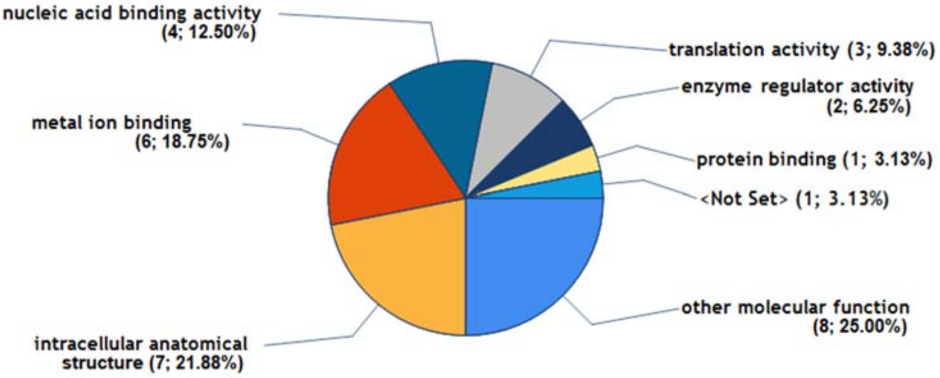

Figure S2. The molecular functions of FLS being altered by C1P in the presence of IL-1ß. The 10 proteins were reproducibly upregulated by at least 1.2-fold or downregulated by 0.8-fold by C1P + IL-1ß in FLS during 48 h of treatment relative to FLS treated with IL-1ß alone. Table S2 provides further data on these proteins. The Go Slim categories for proteins were generated by Proteome Discoverer 2.5 software using the Gene Ontology (GO) database.

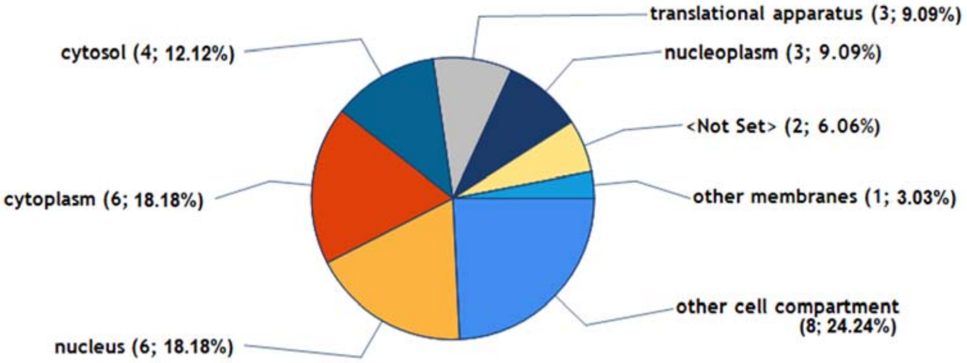

Figure S3. The cellular localization of 10 proteins being regulated by C1P in the presence of IL-1ß. The 10 proteins were reproducibly upregulated by at least 1.2-fold or downregulated by 0.8-fold in FLS by C1P + IL-1ß during 48 h of treatment relative to FLS treated with IL-1ß alone. Table S2 provides further data on these proteins. The Go Slim categories for proteins were generated by Proteome Discoverer 2.5 software using the Gene Ontology (GO) database.

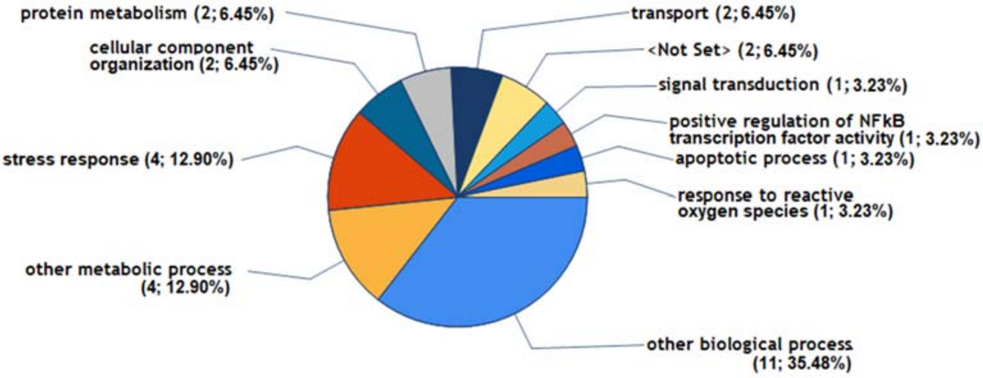

Figure S4. The biological processes of FLS being altered by S1P in the presence of IL-1ß. The 13 proteins were reproducibly upregulated by at least 1.2-fold or downregulated by 0.8-fold in FLS by S1P + IL-1ß during 48 h of treatment relative to FLS treated with IL-1ß alone. Table S2 provides further data on these proteins. The Go Slim categories for proteins were generated by Proteome Discoverer 2.5 software using the Gene Ontology (GO) database.

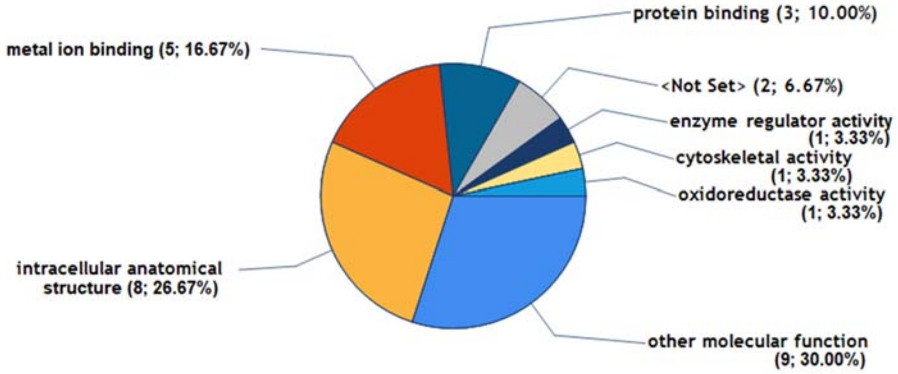

Figure S5. The molecular functions of FLS being altered by S1P in the presence of IL-1ß. The 13 proteins were reproducibly upregulated by at least 1.2-fold or downregulated by 0.8-fold by S1P + IL-1ß in FLS during 48 h of treatment relative to FLS treated with IL-1ß alone. Table S2 provides further data on these proteins. The Go Slim categories for proteins were generated by Proteome Discoverer 2.5 software using the Gene Ontology (GO) database.

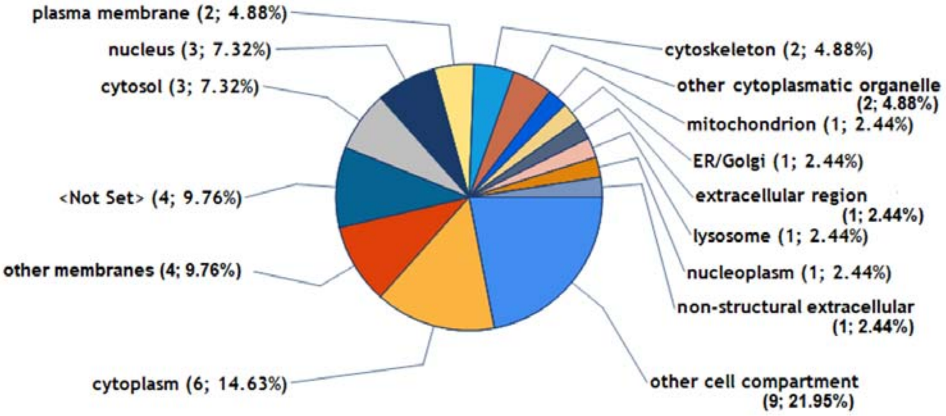

Figure S6. The cellular localization of 13 proteins being regulated by S1P in the presence of IL-1ß. The 13 proteins were reproducibly upregulated by at least 1.2-fold or downregulated by 0.8-fold in FLS by S1P + IL-1ß during 48 h of treatment relative to FLS treated with IL-1ß alone. Table S2 provides further data on these proteins. The Go Slim categories for proteins were generated by Proteome Discoverer 2.5 software using the Gene Ontology (GO) database.

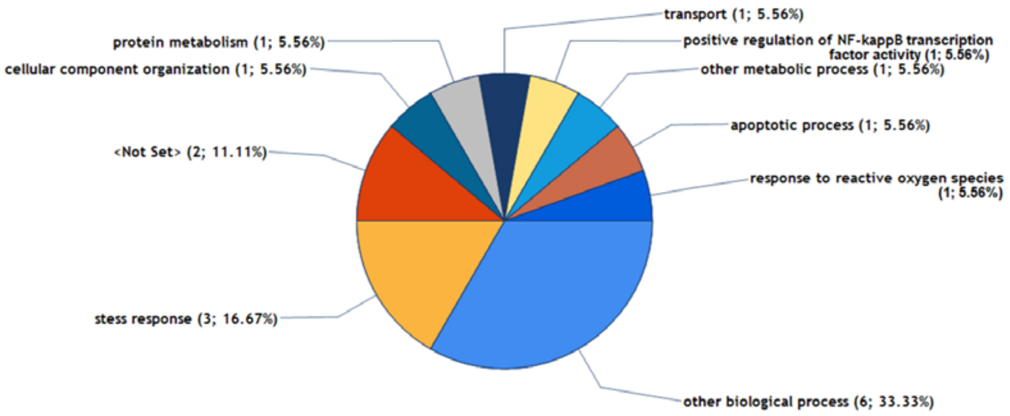

Figure S7. The biological processes of FLS being altered by SPC in the presence of IL-1ß. The 8 proteins were reproducibly upregulated by at least 1.2-fold or downregulated by 0.8-fold in FLS by SPC + IL-1ß during 48 h of treatment relative to FLS treated with IL-1ß alone. Table S2 provides further data on these proteins. The Go Slim categories for proteins were generated by Proteome Discoverer 2.5 software using the Gene Ontology (GO) database.

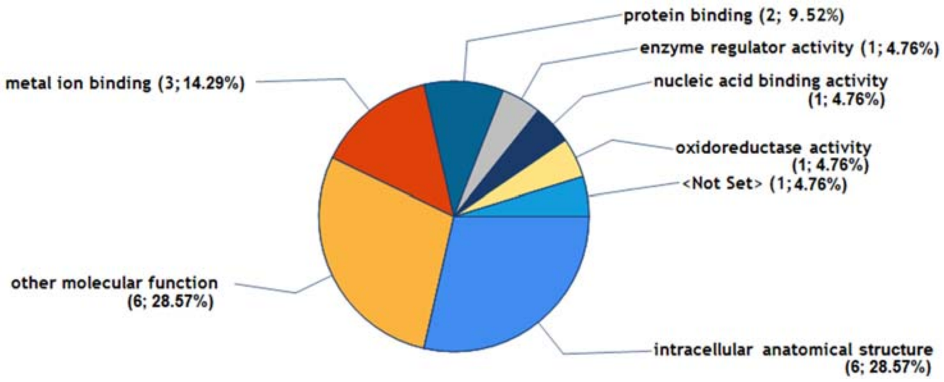

Figure S8. The molecular functions of FLS being altered by SPC in the presence of IL-1ß. The 8 proteins were reproducibly upregulated by at least 1.2-fold or downregulated by 0.8-fold by SPC + IL-1ß in FLS during 48 h of treatment relative to FLS treated with IL-1ß alone. Table S2 provides further data on these proteins. The Go Slim categories for proteins were generated by Proteome Discoverer 2.5 software using the Gene Ontology (GO) database.

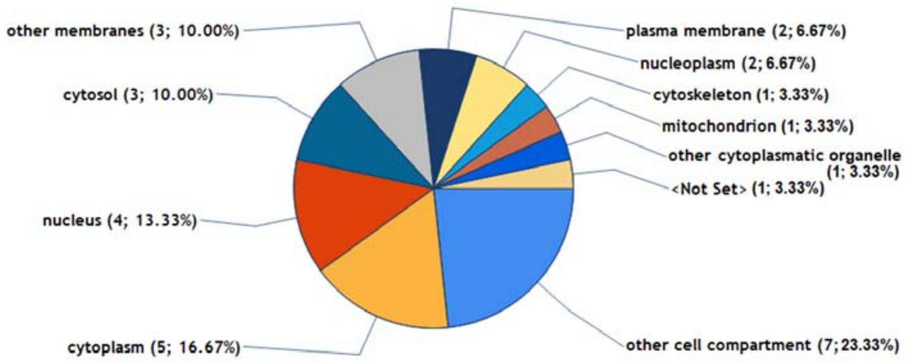

Figure S9. The cellular localization of 8 proteins being regulated by SPC in the presence of IL-1ß. The 8 proteins were reproducibly upregulated by at least 1.2-fold or downregulated by 0.8-fold in FLS by SPC + IL-1ß during 48 h of treatment relative to FLS treated with IL-1ß alone. Table S2 provides further data on these proteins. The Go Slim categories for proteins were generated by Proteome Discoverer 2.5 software using the Gene Ontology (GO) database.
